# Outcome of Tumor-Associated Proptosis in Patients With Spheno-Orbital Meningioma: Single-Center Experience and Systematic Review of the Literature

**DOI:** 10.3389/fonc.2020.574074

**Published:** 2020-10-07

**Authors:** Matthias Schneider, Anna-Laura Potthoff, Valeri Borger, Alexis Hadjiathanasiou, Niklas Schäfer, Ági Güresir, Hartmut Vatter, Ulrich Herrlinger, Erdem Güresir, Patrick Schuss

**Affiliations:** ^1^Department of Neurosurgery, University Hospital Bonn, Bonn, Germany; ^2^Division of Clinical Neuro-Oncology, Department of Neurology, University Hospital Bonn, Bonn, Germany

**Keywords:** spheno-orbital meningioma, skull base surgery, proptosis-outcome, exophthalmos index, review of the literature

## Abstract

**Objective:** Tumor-associated proptosis comprises a frequent phenomenon that negatively impacts quality of life in patients suffering from spheno-orbital meningioma (SOM). Therefore, proptosis outcome represents an important measure in meningioma surgery. In the current study, we analyzed our institutional database in order to evaluate the recovery of tumor-associated proptosis in patients with SOM.

**Methods:** Between 2009 and 2019, 32 patients with SOM underwent surgical treatment at the authors' institution. The exophthalmos index (EI) was calculated by means of preoperative and postoperative tumor-associated proptosis. Patients with preoperative EI ≥ 1.1 were included in further analysis. Further, we performed a systematic review of the contemporary literature. Favorable proptosis outcome was defined as postoperative decreased EI compared with preoperative EI.

**Results:** Overall, 25 of 32 patients with SOM (78%) suffered from preoperative proptosis in the present series. Preoperative mean EI of 1.37 ± 0.18 decreased after surgical treatment to a postoperative mean EI of 1.15 ± 0.1 during follow-up (*p* < 0.0001). Systematic review of the literature revealed three studies with individual data on preoperative and postoperative EI measurements leading to a total of 103 patients; 100 of 103 patients (97%) with SOM and preoperative proptosis achieved favorable outcome.

**Conclusions:** The EI provides a comparable standard in evaluation of surgical outcome in patients with tumor-associated proptosis due to SOMs. The large dataset consisting of pooled individual patient data from the systematic review of the literature and the present case series support the assumption that surgical treatment is highly effective in the treatment of tumor-associated proptosis in SOM.

## Introduction

Tumor-associated proptosis is a typical presenting symptom in patients suffering from spheno-orbital meningiomas (SOMs) (1, 2). In patients with SOM or other skull base meningiomas, proptosis is often perceived as cosmetically and/or functionally attenuating (2). Despite microsurgical resection representing the standard treatment modality for clinically manifest meningiomas, several previous reports have discussed the optimal surgical approach, extent of resection, and the need for orbital reconstruction (2–5). However, standardized evaluation and comparability of initial characteristics and postoperative outcome of tumor-associated proptosis were cumbersome until the implementation of the exophthalmos index (EI) by Scarone et al. in 2009 (1). Therefore, patient data that enable a robust comparability of surgical results in the case of tumor-associated proptosis in patients with SOM are scarce.

Therefore, the objective of the present study was not only to add comparable data on proptosis outcome after surgical treatment of SOM but also to enable comparison by individual patient data extraction and pooling from a systematic review of the literature leading to the largest comparable dataset on proptosis in patients suffering from SOMs.

## Methods

### Patients

Between May 2009 and September 2019, 32 patients with SOM aged 18 years or older were surgically treated at our institution. Review of records was performed retrospectively after institutional review board approval had been obtained. Pertinent clinical information including age, sex, Karnofsky Performance Scale (KPS), tumor localization, tumor size, and presence of peritumoral edema, WHO grade referring to postoperative histological examination, extent of tumor resection according to the Simpson grading system, presence of preoperative visual symptoms, and presence and value of preoperative and postoperative proptosis were collected and entered into a computerized database (SPSS, version 25, IBM Corp., Armonk, NY). Furthermore, presence and value of preoperative and postoperative proptosis were independently analyzed by two authors (A-LP and PS). No disagreements were found. In addition, postoperatively worsened or newly diagnosed cranial nerve morbidity assessed at the 6-months follow-up examination as well as postoperative cerebrospinal fluid (CSF) leakage with insertion of a lumbar drainage system and/or secondary implantation of a ventriculoperitoneal shunt system as perioperative and postoperative complications was recorded.

Histopathological grading was performed according to the 2016 WHO criteria (6). All previous pathology reports underwent renewed review to confirm that diagnosis was in accordance to these requirements. Patients underwent standardized preoperative clinical, ophthalmological, computed tomography (CT), and magnetic resonance imaging (MRI) examinations. Clinical and imaging follow-up consisted of MRI scans 3 months after surgery as well as a yearly imaging for the following 5 years. Earlier clinical and imaging evaluation was advised in case of new or worsened neurological deficits as well as radiological signs of tumor recurrence or progression.

**Figure 1:**
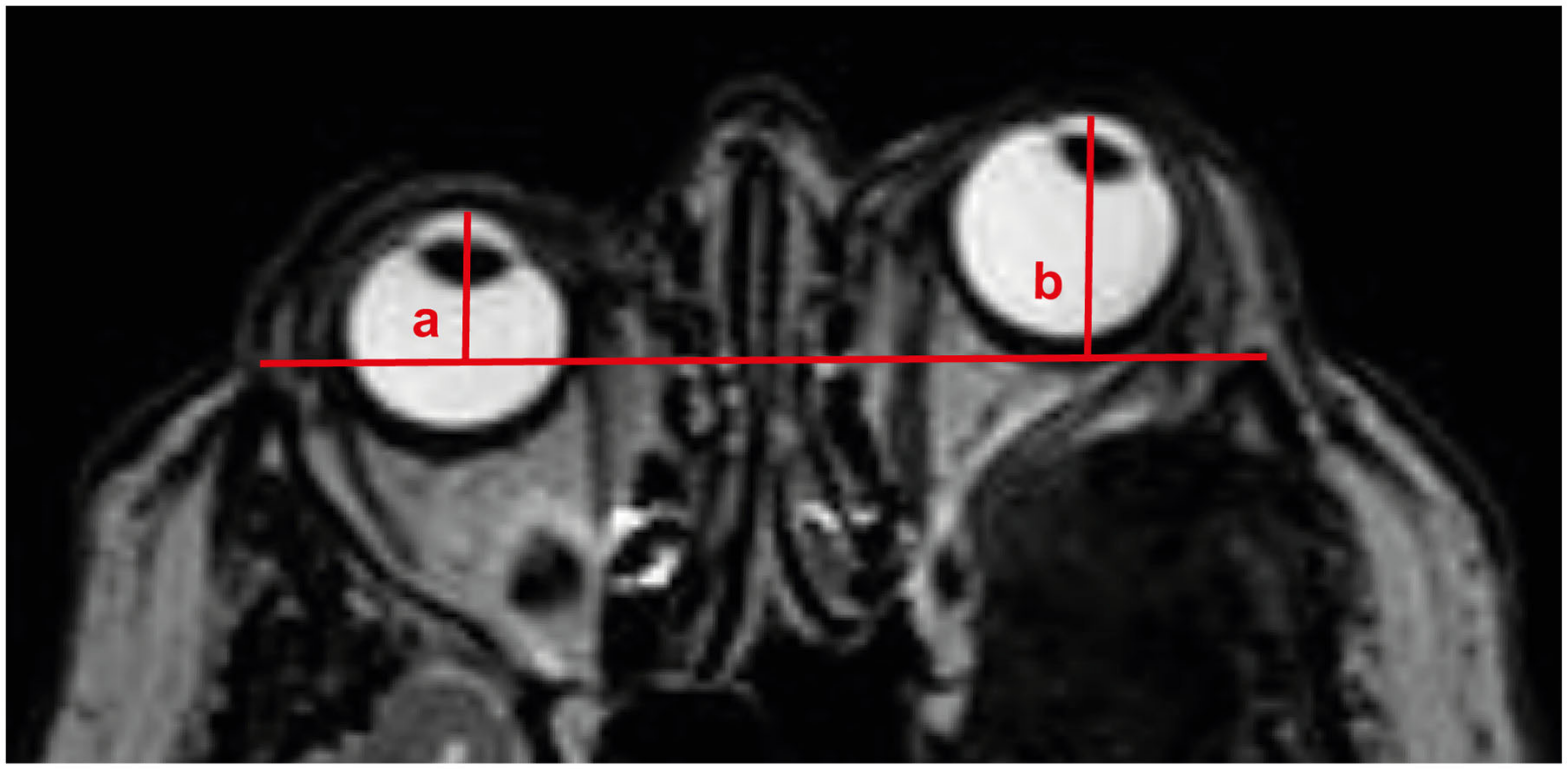
llustration of preoperative calculation of exophthalmos indices. The distance of the anterior limit of each eye globe to a line between both anterior margins of the frontal processes of the zygomas is measured. EI is calculated as the distance ratio between the pathological eye and the normal eye (b/a = 22.8 mm/15.3 mm = 1.5 for the presented case). EI, exophthalmos index.

Preoperative and postoperative tumor-associated proptosis was measured by the EI as previously described by Scarone et al. (1). Therefore, a line between both anterior margins of the frontal processes of the zygomas has to be drawn. Afterwards, the distance of the anterior limit of each eye globe to this line is measured, comparing the pathological eye with the unimpaired eye (Figure 1). Symmetric position of both ocular globes correlates to an EI of exactly 1.0, with EI > 1.0 indicating proptosis. In order to reduce potential measurement inconsistency, cases of preoperative EI < 1.1 were excluded from further analysis.

### Surgical Approach and Orbital Reconstruction

The surgical approach consisted of frontolateral or pterional craniotomy with removal of the hyperostic bone of the lateral orbital wall. Depending on the bone infiltration caused by SOM, the orbital roof or the zygoma was partly removed. According to the treating neurosurgeon decision, an anterior clinoidectomy with unroofing of the optic canal was performed. Tumor extensions in the cavernous sinus were usually spared in order to obviate postoperative new neurological deficits. In cases of intraorbital tumor infiltration, resection was carried out with particular care for intraorbital anatomical structures.

**Figure 2:**
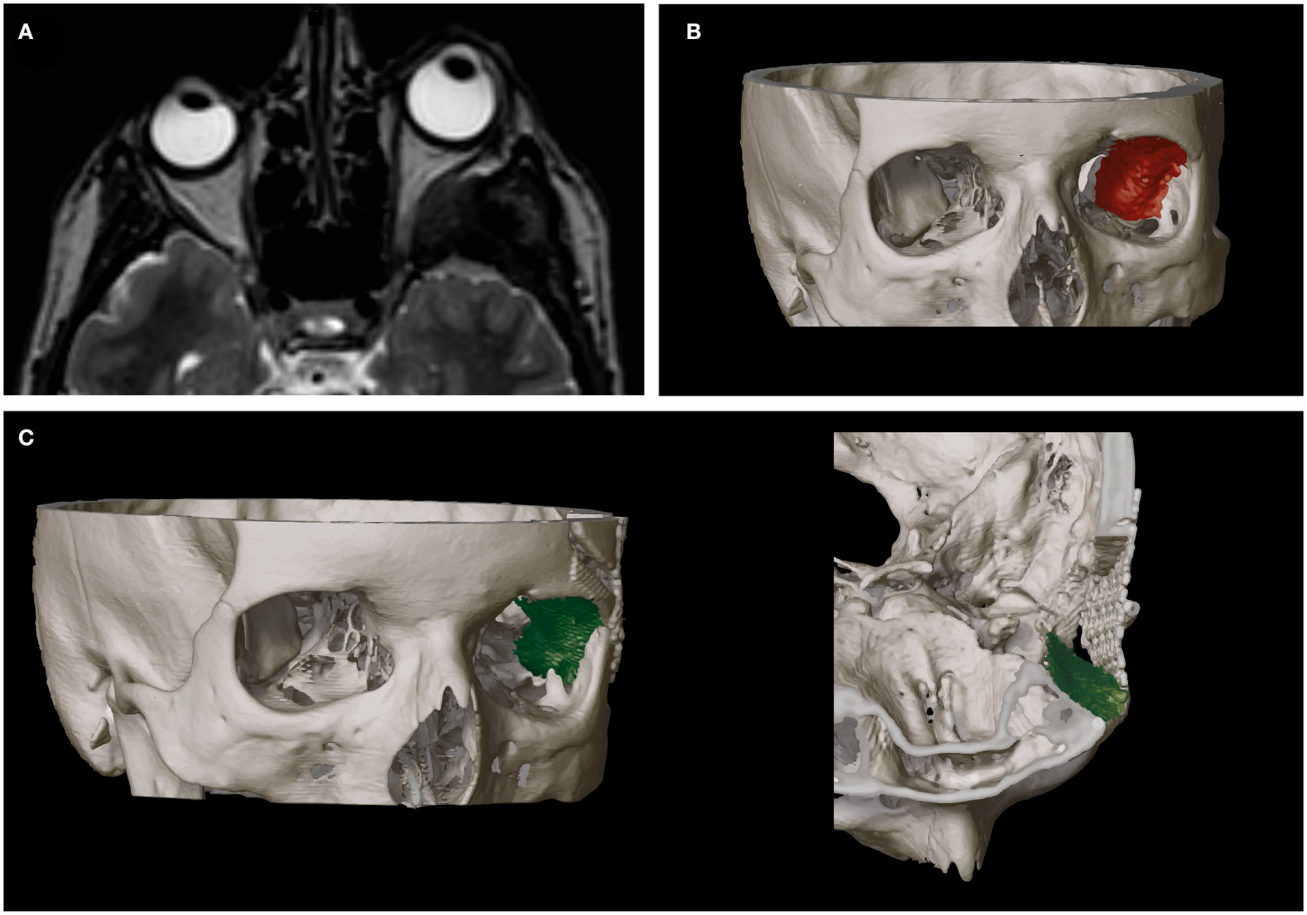
Orbital reconstruction after removal of hyperostotic lateral orbital bone enables restoring of physiological intraorbital anatomy. Illustration of preoperative proptosis of the left eyeball **(A)** as a result of meningioma-induced lateral orbital wall hyperostosis (red) **(B)**. **(C)** Surgical reconstruction of the lateral orbital wall (green) yields removal of intraorbital space-occupying effects and restores intraorbital physiological topography (left, fronto-temporal view; right, cranial view).

Lateral and superior orbital walls were reconstructed to fit the anatomically normal structure for each patient using intraoperative navigation guidance. Orbital and sphenoid wing reconstruction was performed with titanium mesh in all patients (Figure 2).

### Systematic Review

#### Search Methods

In order to gain a larger population, we performed a systematic review of the literature using the MEDLINE database (latest access February 2020). The following keywords were queried individually or in relevant combinations: “spheno-orbital meningioma,” “exophthalmos,” and “proptosis.” Full-text versions were obtained from all studies that were independently reviewed and considered to be relevant by two authors (MS and A-LP). Any disagreement between the two authors was resolved in consensus meetings with the senior author (PS). References of relevant studies were searched for additional articles of interest.

#### Selection Criteria

We analyzed studies of patients suffering from SOM with tumor-associated proptosis as well as their references. Articles were included when they analyzed and reported detailed individual data on preoperative and postoperative proptosis. Only studies using the EI to quantify tumor-associated proptosis were included in order to increase data comparability.

Anecdotal single case reports and case series with detailed individual data exclusively provided in a limited number of patients were excluded in order to reduce potential super-selection bias.

##### Data Collection and Extraction

We extracted data on patient characteristics, preoperative EI, presence of visual symptoms, surgical reconstruction technique, Simpson grade, WHO grade, postoperative EI, and postoperative visual outcome. Proptosis outcome was stratified by the reported clinical status at the last follow-up into favorable (difference between preoperative and postoperative EI > 0) vs. unfavorable (difference between preoperative and postoperative EI < 0). Data were independently extracted and verified by two authors (MS and A-LP). No disagreements were found.

### Statistics

Data analyses were performed using the computer software package SPSS (version 25, IBM Corp., Armonk, NY). The D'Agostino–Pearson test was used to quantify deviations from normal distribution. In the case of *p* < 0.05, Wilcoxon matched-pairs signed rank test was performed. Results with *p* < 0.05 were considered statistically significant. Violin plots were programmed using R-software.

## Results

### Present Series

Overall, 32 patients with SOM were treated surgically at our institution from May 2009 until September 2019; 25 of 32 patients (78%) suffered from tumor-associated proptosis with an EI > 1.1 and were therefore included in further analysis. Mean patient age was 58 ± 12 years. Simpson grade I resection was achieved in six patients (24%), Simpson grade II in 12 patients (48%), and Simpson grade III and IV resection of SOM in seven patients (28%) with tumor-associated proptosis. Histopathological assessment revealed WHO grade I tumors in 22 patients (88%), whereas three patients (12%) suffered from WHO grade II meningiomas. The median follow-up time from surgical treatment to last follow-up was 55 months. Tumor recurrence was present in four patients (16%) with one subject following Simpson grade II and III resections and two subjects following Simpson grade IV resections. Retreatment consisted of adjuvant radiotherapy in four cases (16%). Further details on patient and tumor characteristics are given in Table 1. Postoperative new or worsened cranial nerve deficits examined 6 months after surgery were present in nine of 25 patients (36%) with SOM and tumor-associated proptosis. Thereby, cranial nerves II and III were the most affected cranial nerves accounting for three (12%) and six (24%) cases, respectively. Postoperative CSF leakage was present in two of 32 patients (6%) with secondary shunt dependency in one subject (3%).

**Table 1:** Patient characteristics in present series.

**Case no**.	**Age (years), sex**	**Tumor WHO grade**	**Simpson grade**	**Cavernous sinus infiltration**	**Preoperative EI**	**Postoperative EI**	**Adjuvant RTX**	**Recurrence**	**FU (months)**
1	53, M	I	II	Yes	1.16	1.05	No	No	23
2	52, M	II	II	No	1.25	1.04	No	No	104
3	60, F	I	II	No	1.28	1.15	No	No	89
4	51, F	I	IV	Yes	1.24	1.05	No	No	55
5	57, F	II	III	Yes	1.47	1.10	Yes	No	78
6	49, F	I	II	No	1.38	1.03	No	No	27
7	64, F	I	II	No	1.38	1.26	Yes	Yes	15
8	59, F	I	IV	Yes	1.49	1.15	No	No	12
9	66, F	I	II	No	1.17	1.03	No	No	7
10	73, F	I	I	No	1.32	1.06	No	No	107
11	78, F	I	I	No	1.37	1.25	No	No	102
12	48, F	I	I	No	1.68	1.16	No	No	99
13	39, F	I	I	Yes	1.29	1.09	No	No	99
14	29, F	I	IV	Yes	1.77	1.41	Yes	Yes	91
15	61, F	I	I	No	1.34	1.14	No	No	86
16	69, F	I	IV	No	1.77	1.39	No	Yes	82
17	79, F	I	II	No	1.20	1.11	No	No	80
18	50, F	I	II	No	1.22	1.18	No	No	77
19	69, M	I	II	No	1.31	1.19	No	No	53
20	57, F	I	I	No	1.40	1.18	No	No	53
21	52, F	I	II	No	1.34	1.13	No	No	49
22	67, F	I	II	No	1.21	1.08	No	No	38
23	56, F	I	IV	Yes	1.27	1.12	Yes	Yes	14
24	68, F	II	IV	Yes	1.68	1.23	No	No	6
25	42, F	I	II	Yes	1.24	1.10	No	No	26

*WHO, World Health Organization; EI, exophthalmos index; RTX, radiotherapy; FU, follow-up; M, male; F, female*.

#### Exophthalmos Index

**Figure 3:**
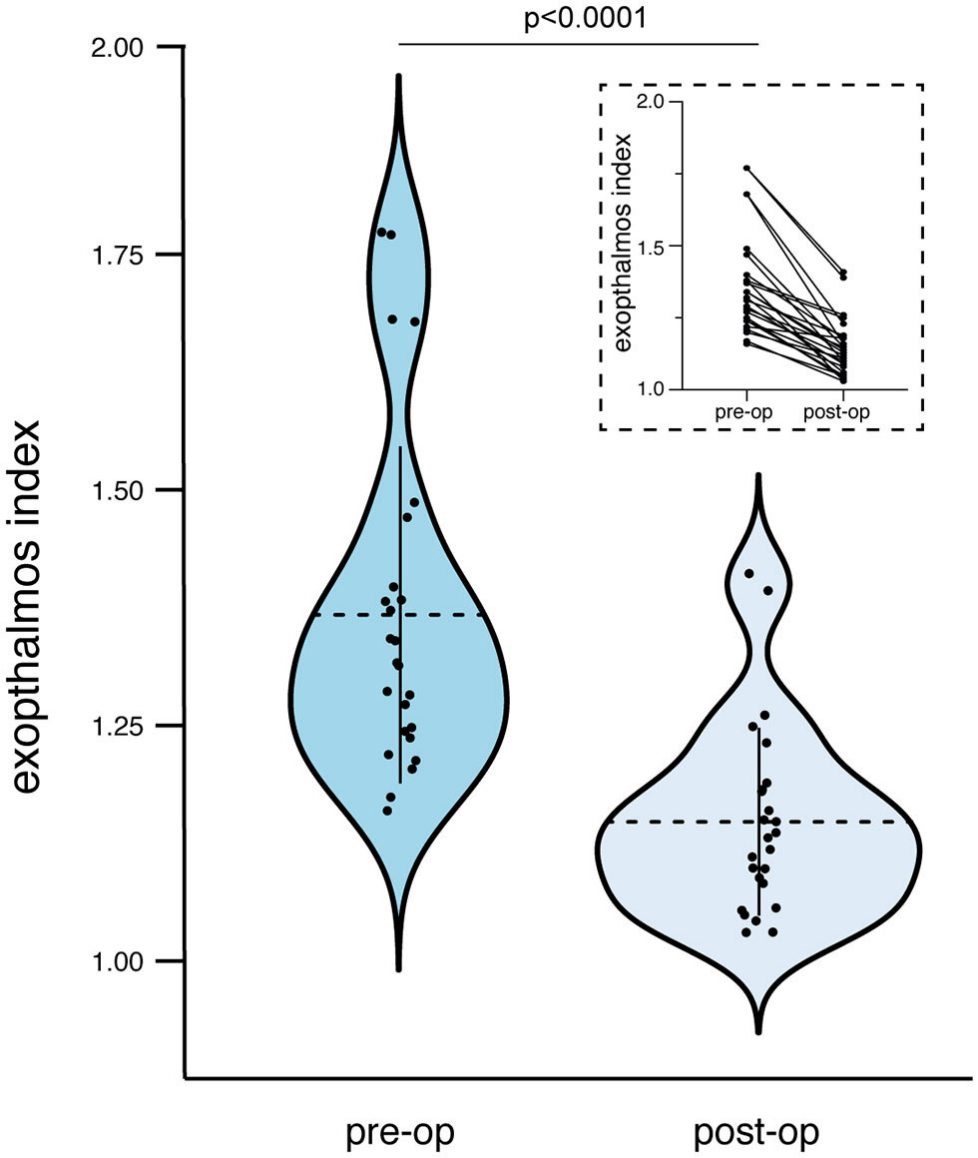
Violin and before-after plots depicting resolution of proptosis following resection of spheno-orbital meningioma (present series). Violin plot shows mean and distribution of preoperative and postoperative EI, whereas before-after plot illustrates the difference between initial and follow-up EI for each patient individually. EI, exophthalmos index.

Patients with tumor-associated proptosis presented in the current series with an initial mean EI of 1.37 ± 0.18. After surgical treatment, the postoperative mean EI in those patients after 6 months or at last follow-up was 1.15 ± 0.1. This results in a mean difference between initial and follow-up EI of 0.22 ± 0.12. Preoperative extent of proptosis was significantly distinct compared with the postoperative results after surgical treatment of SOM (*p* < 0.0001, CI 95% 0.17–0.27; Figure 3). Both Simpson grade I and II resections as an aggressive meningioma resection regime and Simpson grade III and IV resections as a rather meningioma mass reduction policy revealed a significant decrease of preoperative extent of proptosis [preoperative and postoperative mean EI of 1.52 (CI 95% 1.32–1.73) and 1.21 (CI 95% 1.08–1.34) for Simpson grades I and II, *p* = 0.016; respective values for Simpson grades II and IV were 1.31 (CI 95% 1.25–1.37) and 1.12 (CI 95% 1.09–1.16), *p* < 0.0001].

### Search Result

**Figure 4:**
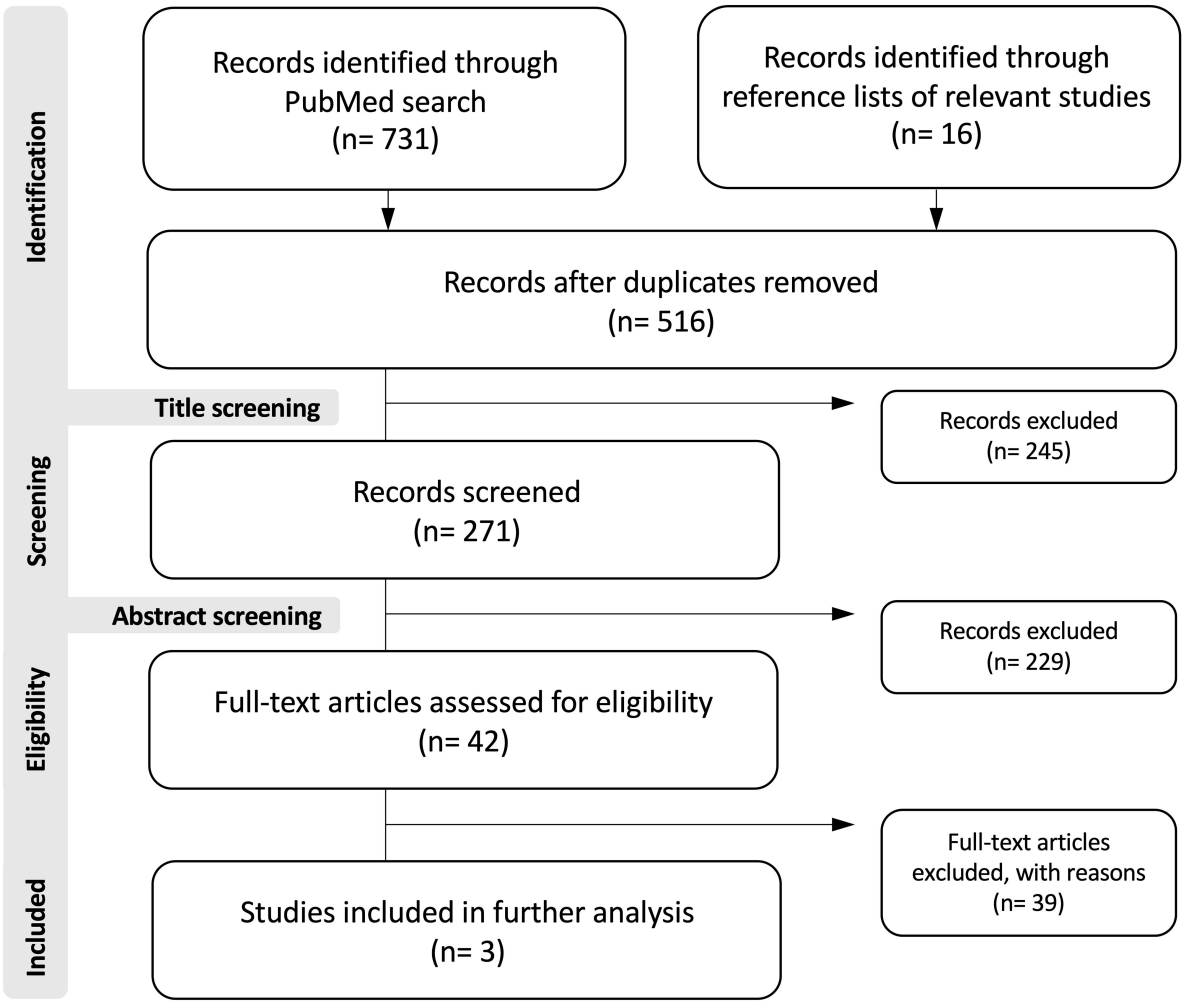
Flowchart depicting the search strategy.

The MEDLINE search yielded a total of 731 titles, of which 42 were considered relevant after filtering duplicates and application of our above-mentioned selection criteria. After review of the remaining articles, three studies reporting on a total of 78 patients met the inclusion criteria (Figure 4) (1, 2, 7). All included articles were classified as retrospective case series. Together with the current series of 25 patients with SOM and tumor-associated proptosis, there were a total of 103 patients included in the pooled dataset. Patient characteristics of the pooled data are detailed in Table 2.

**Table 2:** Systematic review on proptosis outcome following spheno-orbital meningioma resection.

**Authors and year**	**Patients included in further analysis**	**Mean preoperative EI ± SD**	**Mean postoperative EI ± SD**	**Patients with favorable proptosis outcome (%)**
Scarone et al., 2009 (1)	30	1.88 ± 0.44	1.51 ± 0.37	29 (97)
Bowers et al., 2016 (2)	32	1.39 ± 0.28	1.13 ± 0.14	32 (100)
Freeman et al., 2017 (7)	16	1.33 ± 0.17	1.15 ± 0.13	14 (88)
present series, 2020	25	1.37 ± 0.18	1.15 ± 0.09	25 (100)
Total	103	1.52 ± 0.38	1.25 ± 0.28	100 (97)

*EI, exophthalmos index; SD, standard deviation*.

#### Influence of Surgical Treatment on Proptosis

Overall, 100 of 103 patients (97%) achieved favorable proptosis outcome after surgical treatment of SOM. In detail, patients with SOM and tumor-associated proptosis in the pooled dataset presented with an initial mean EI of 1.52 ± 0.38. After surgical treatment, the postoperative mean EI in those patients after the last follow-up reported in the selected studies was 1.25 ± 0.28. This results in a mean difference between initial and last reported follow-up EI of 0.27 ± 0.26. Therefore, in the pooled data, preoperative extent of proptosis was significantly distinct as compared with the postoperative results after surgical treatment of SOM (*p* < 0.0001, CI 95% 0.18–0.4; Table 2).

## Discussion

Tumor-associated proptosis represents common concomitant impairment in patients with SOM. Despite cosmetic issues, certain impairment of functionality might result from tumor-associated proptosis (8). However, a rising number of reports stated that surgical treatment of SOM influences favorable outcome of tumor-associated proptosis in multiple fashion.

### Reconstruction of the Orbit

Surgical reconstruction of the orbit in patients with SOM is still a controversially discussed topic. Heller et al. counteracted several considerations concerning the orbital wall reconstruction due to discussing the influence of the overall orbital volume after reconstruction (8). Heller et al. suggested three potential considerations with (a) smaller orbital reconstruction due to previous chronic compression and fat necrosis leading to a smaller orbital volume, (b) larger orbital reconstruction in order to prevent postoperative scar tissue to impair venous drainage from the orbita, and (c) orbital volume reconstruction estimated as anatomically normal for each patient (8). Furthermore, multiple techniques and materials for orbital reconstruction have been described previously (3, 4, 9).

In cases of absent orbital reconstruction, risk of postoperative development of pulsatile enophthalmos, meningoceles, diplopia, and extraocular muscle fibrosis leading to ophthalmoplegia should be often remembered (7, 10, 11). However, several groups reported their experience on improvement of tumor-associated proptosis after no orbital reconstruction was performed after surgery leading to satisfactory cosmetic results, and they pointed out the above-mentioned complications must not necessarily result (7, 10).

In the present study, all patients underwent rigid individual orbital reconstruction of the lateral orbital wall using titanium mesh leading to favorable proptosis outcome.

### Change in Surgical Strategy

Meanwhile, before controversial discussions of orbital reconstruction methods and needs, the surgical strategy in patients suffering from SOM itself was the subject of several arguments. Previously, aggressive tumor excisions including the resection of the dural tail providing the best tumor control rates were postulated. However, concerning the location of SOM and delicate structures of the orbital cone, radical resection might facilitate postoperative complications (1, 4). Furthermore, previous reports stated a high level of new cranial nerve morbidity after radical removal of frontal skull base meningiomas in previous decades (12–14). Ringel et al. reported 30% new cranial nerve deficits after surgical resection of SOM in a large series of patients treated from 1983 to 2006 (4). Therefore, a recent shift from aggressive surgical therapy toward a symptom-oriented surgery has witnessed symptom-oriented surgery in patients with SOM, mainly with focus on optic nerve decompression or treatment of proptosis (1, 5). The present series confirmed the rationale behind this paradigm shift by revealing profound reduction of preoperative EI in both case of aggressive Simpson grade I/II and Simpson grade III/IV resections as rather decompressive resection regimens.

The most recent studies disprove the assumption that only initial radical resection of SOM enhanced long-term tumor control in these patients (1, 4, 15). Long-term surveillance with constant follow-up consultation seems the widely accepted monitoring method in patients with SOM with repeated surgery when tumor recurrence causes cranial nerve deficits, such as visual symptoms (1, 11, 16).

Further, the symbiotic role of postoperative radiotherapy is evolving (17). In patients with higher WHO grading and/or necessity of rigorous tumor control, radiosurgical treatment after partial resection and surgical decompression of essential intracranial structures is increasingly advocated by several authors (15, 17). In the present series, successful orbital decompression indicated by sufficient decrease in preoperative EI was followed by postoperative radiotherapy in four patients with recurrent meningioma. Thus, with regard to adjuvant secondary treatment modalities, aggressive meningioma excisions in high-risk areas for increased postoperative morbidity such as the spheno-orbital region hardly seem to be justified with regard to an improvement in the rate of postoperative tumor recurrence.

### Resolution of Proptosis After Surgical Treatment

Postoperative results of tumor-associated proptosis are inconsistently reported throughout the literature with mostly reporting on proptosis improvement in qualitative terms that do not entirely reflect the individual variability in ocular globe position (1, 2, 8). The number of studies investigating the influence of surgical treatment on correction of tumor-associated proptosis in a quantifiable fashion is limited (1, 2, 8). Therefore, Scarone et al. established the EI in 2009 (1). The EI is a simple tool producing reliable data that can be compared across different studies (8). Due to this previously mentioned comparability, we performed a systematic review of the literature, extracted individual patient data meeting our inclusion criteria, and gained the largest comparable patient dataset concerning resolution of tumor-associated proptosis after surgery for SOM. The results of our own present series are in line with those of the literature. The pooled data with a favorable proptosis outcome in 97% of the treated patient led to the assumption that surgical treatment of tumor-associated proptosis is promising. However, more studies are desirable, which present comparable data by the use of EI measurements for further and detailed comparison of patients with SOM regarding the different reconstruction and treatment strategies.

## Limitations

The present study has several limitations. Acquisition of data was retrospective. Patients were not randomized but treated by the preference of the treating. However, the use of the EI as measuring instrument enables reliable and quantitative assessment of proptosis. The limited number of studies reporting data on EI and SOM nevertheless presented individual patient data, which allowed qualitative data pooling and therefore establishment of a large patient dataset for further analysis. However, the results of the present pooled dataset should engage further prospective study of SOM regarding surgical techniques as well as quantitative proptosis outcome.

## Conclusions

The EI provides a comparable standard in evaluation of surgical outcome in patients with tumor-associated proptosis due to SOMs. The large dataset consisting of pooled individual patient data from the systematic review of the literature and the present case series supports the assumption that surgical treatment is highly effective in the treatment of tumor-associated proptosis in SOM.

## Data Availability Statement

The raw data supporting the conclusions of this article will be made available by the authors, without undue reservation.

## Ethics Statement

The studies involving human participants were reviewed and approved by Local Ethics Committee at the University Hospital Bonn. Written informed consent for participation was not required for this study in accordance with the national legislation and the institutional requirements. Written informed consent was not obtained from the individual(s) for the publication of any potentially identifiable images or data included in this article.

## Author Contributions

Conceptualization was performed by MS, PS, and UH. MS, A-LP, and PS performed the methodology and statistical analysis. Data collection was performed by A-LP, VB, and AH. Figures were provided by MS, A-LP, and PS. MS and PS wrote the original draft and supervised the study. Proof-reading was done by MS, A-LP, NS, ÁG, HV, EG, and PS.

## Conflict of Interest

The authors declare that the research was conducted in the absence of any commercial or financial relationships that could be construed as a potential conflict of interest.
